# How big data analytics can strengthen large‐scale food fortification and biofortification decision‐making: A scoping review

**DOI:** 10.1111/nyas.70028

**Published:** 2025-09-10

**Authors:** Fiona Walsh, Anna Zhenchuk, Corey Luthringer, Christoph Kratz, Florian Schweigert

**Affiliations:** ^1^ Heidelberg Institute of Global Health (HIGH), Medical Faculty and University Hospital Heidelberg University Heidelberg Germany; ^2^ Mesurado Cooperative Boston Massachusetts USA; ^3^ BioAnalyt Teltow Germany; ^4^ Department of Physiology and Pathophysiology Institute of Nutritional Science University of Potsdam Potsdam Germany

**Keywords:** artificial intelligence, big data analytics, biofortification, food value chain, large‐scale food fortification, machine learning, nutrition

## Abstract

Big data analytics have shown great potential to improve decision‐making in health, including disease surveillance and healthcare delivery. This scoping review explores how big data supports decision‐making in large‐scale food fortification (LSFF) and biofortification across the food value chain. Following PRISMA guidelines, we analyzed open‐access peer‐reviewed literature and gray literature from 2012 to 2022. Given the limited literature, we broadened our search to include big data applications in agriculture and nutrition, aiming to draw relevant insights for LSFF and biofortification. Of 1678 records, 28 mentioned LSFF or biofortification, all published between 2018 and 2022. Overall, most records focused on production (60%) and inputs (19.5%). Notably, 16.7% (*n* = 7) of records mentioning LSFF or biofortification addressed public health monitoring, compared to 2.3% (*n* = 45) of those without a mention. Use case examples include blockchain and Internet of Things (IoT) for fortified product traceability, machine learning to predict fortification gaps, and artificial intelligence to analyze anemia prevalence, highlighting opportunities to enhance both production and public health monitoring. Despite this potential, big data use in LSFF and biofortification remains limited. Expanding its use in underexplored areas, such as distribution and regulation, could enhance decision‐making, efficiency, and sustainability in LSFF and biofortification.

## Graphical abstract

This scoping review explores the use of big data analytics to support decision‐making in large‐scale food fortification (LSFF) and biofortification across the food value chain.

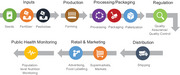

## INTRODUCTION

Hidden hunger affects more than two billion people globally, particularly in low‐ and middle‐income countries (LMICs), where diets are heavily reliant on staple foods that fail to provide the recommended daily intake of essential micronutrients.^1^, ^2^ Micronutrient deficiencies (MNDs) disproportionately impact young children and women of reproductive age, contributing to adverse health outcomes and undermining global progress toward the Sustainable Development Goals.^3^, ^4^

Large‐scale food fortification (LSFF) and biofortification are among the most cost‐effective measures to prevent MNDs.^5^ LSFF involves adding micronutrients to widely consumed foods during processing, while biofortification enhances the nutrient content of food crops during their growth through plant breeding or mineral fertilization.^6^ Both strategies face distinct challenges. LSFF programs often struggle with industry compliance, regulatory monitoring, and persistent under‐fortification in some regions, while biofortification requires widespread adoption by farmers and long‐term sustainability through continued crop development and market integration.^7^, ^8^, ^9^

While LSFF and biofortification are widely recognized as strategies for reducing MNDs, there remains a need for high‐quality data on micronutrient intake and consumption patterns to optimize these interventions. These data are critical for determining what micronutrients to add, to which foods, and in what quantities.^10^ However, available data are often fragmented, outdated, or nonstandardized, limiting their usefulness in informing policy decisions and LSFF and biofortification strategies.^10^ This gap in high‐quality data undermines policymakers’ ability to target MND interventions where they are most needed, reducing the overall impact and cost‐effectiveness of these fortification strategies.

At the same time, the rise of Industry 4.0—the Fourth Industrial Revolution—is driving disruptive technological innovation across many sectors, including finance, health, and energy.^11^ By integrating vast amounts of data, commonly known as big data, manufacturers can leverage artificial intelligence (AI) and machine learning to automate and optimize analytics processes, revealing transformative business insights.^12^, ^13^, ^14^, ^15^ Big data refers to a large, complex dataset that combines multiple variables, making them challenging to analyze using conventional statistical methods.^16^ The most widely referenced definition of big data is the 5‐V model, which highlights five key characteristics: high volume (a large amount of data, often exceeding tera‐ or petabytes), high velocity (fast speed of data generation such as near real‐time streaming data), high variety (many diverse data formats and structures from multiple sources), high veracity (conformity with facts and closely related to data quality), and high value (the derived information provides more benefits and insights to decision‐makers than traditional data sources).^17^

Despite the vast amount of data generated across the global food system, the agriculture and nutrition sectors have been slow to harness Industry 4.0 technologies, such as big data and AI, which can identify supply chain inefficiencies, optimize crop management, and provide insights into dietary patterns.^18^ In the context of LSFF and biofortification, big data holds significant promise for enhancing the efficiency, effectiveness, and sustainability of these programs. For instance, technologies such as connected dosifiers can collect real‐time data on the addition of micronutrient premixes to staple foods.^19^ Machine learning approaches can predict nutrient content in fortified products on a large scale, and public health planners can combine data mining with machine learning to analyze dietary data from social media and assess community‐level outcomes of LSFF and biofortification initiatives.

While these applications hold significant potential, we are unaware of any comprehensive review of how stakeholders have utilized big data analytics in LSFF and biofortification programs to date. This study aims to identify the promises and challenges of big data approaches in agriculture and nutrition, particularly in LSFF and biofortification. Furthermore, it explores how stakeholders can apply these insights to improve the efficiency and effectiveness of LSFF and biofortification programs while addressing existing limitations and considering future perspectives. The research question guiding this scoping review is: How is big data used for decision‐making in food fortification across the following dimensions: inputs, production, processing and packaging, distribution, regulation, retail and marketing, and nutrition public health monitoring?^a^

## MATERIALS AND METHODS

### Rationale for scoping review methodology

A scoping review was chosen as the most appropriate methodology for this study because it allows for the systematic mapping of a broad and diverse body of literature on big data applications in LSFF and biofortification.^20^ This topic spans multiple disciplines, including nutrition, agriculture, public health, and data science, and the evidence base is fragmented and heterogeneous, with no prior comprehensive synthesis available. Scoping reviews are particularly useful in such contexts where the aim is not to evaluate the quality or effectiveness of interventions but rather to explore the extent, range, and nature of research activity, clarify key concepts, and identify knowledge gaps in the literature.^20^, ^21^

By using a scoping review approach, we were able to capture a wide array of peer‐reviewed and gray literature, encompassing varied study designs and data sources, and to examine how big data is being applied (or not) across different segments of the food value chain. This method also allowed us to capture emerging applications and innovations that have not yet been systematically examined in the peer‐reviewed literature. Ultimately, the scoping review provided a structured foundation for identifying underexplored areas, such as the limited use of big data in regulation and distribution within LSFF and biofortification, and highlighted opportunities for future research, policy development, and investment.

### Protocol and registration

This scoping review followed the PRISMA‐ScR guidelines (see Appendix S1 in Supplementary Material). We developed the protocol in advance to ensure consistency in the search strategy, study selection, and data extraction, aligning with scoping review best practices.

### Eligibility criteria

We developed eligibility criteria to include records that explicitly mentioned big data, machine learning, AI, or related analytics tools in the context of LSFF, biofortification, or broader food systems along the food value chain. The rationale for these criteria was to ensure the inclusion of relevant and recent literature while excluding unrelated or outdated materials.

We included records if they were: openly accessible; explicitly focused on big data tools or methods applied to the food value chain; and published in peer‐reviewed journals or identified as gray literature from reputable sources (e.g., organizational reports, industry documents).

We excluded records if they focused solely on predictive modeling without large datasets; addressed topics outside the scope of big data or food systems, such as wearable devices, renewable energy, or animal studies; and were published in languages other than English or before 2012. There has been significant growth in new platforms, tools, and methodologies for big data within the last 10 years, which is why this scoping review was limited to studies published after 2012.^22^

### Information sources

The two separate search strategies were conducted on November 28, 2022.

Approach 1. Review of peer‐reviewed publications. Comprehensive searches were conducted across five databases (PubMed, ScienceDirect, Cochrane Database of Systematic Reviews, SciELO, and Google Scholar). There were no restrictions on the type of study design. We searched these databases by reviewing the titles, abstracts, and keywords of the records. In Google Scholar, we reviewed the first five pages of results.

Approach 2. Review of gray literature to identify potentially relevant reports. We may have missed some materials in Approach 1 because they do not match our search terms in the title or abstract of the publication. Therefore, we also manually added unique materials published on the websites of organizations working on LSFF or biofortification, or obtained through a standard Google search. A complete list of gray literature sources and databases is provided in Appendix S2 in the Supplementary Material.

### Search strategy

We designed the search strategy to capture the intersection of big data analytics and the food value chain. Keywords combined terms such as “big data,” “machine learning,” “artificial intelligence,” and “data mining” with dimensions of the food value chain, including “fortification,” “biofortification,” “nutrition,” “processing,” and “monitoring.” A detailed description of the search terms and strategies for each database is included in Appendix S2.

To address the anticipated limitations in the availability of literature on big data applications in food fortification, we expanded our search scope to include broader applications of big data in agriculture and nutrition along the food value chain. This expanded scope aimed to uncover transferable insights that could inform LSFF and biofortification practices, providing a more comprehensive understanding of the role of big data in the broader context of food systems.

### Selection of sources of evidence

One reviewer (F.W.) downloaded all citations from the research databases into EndNote, organized them into a dedicated folder, and then uploaded them to Covidence, a web application used for collaborative systematic reviews. Duplicate records and non‐English studies were excluded by one reviewer (F.W.). Two reviewers (F.W. and F.S.) independently and blindly screened all records’ titles, abstracts, and full texts for eligibility, with reasons for inclusion or exclusion recorded in Covidence. Disagreements were discussed, and any discrepancies were resolved by one reviewer (F.W.). We exported the final list of included and excluded studies, along with the reasons for exclusion, to Microsoft Excel.

### Data charting process

Data charting was conducted using a structured form developed in Microsoft Excel. Variables charted included publication year, region of focus, data sources, and the type of analytics methodology used. Two reviewers (F.W. and F.S.) independently extracted the data and validated the results through team discussions to ensure consistency.

### Data items

Finally, we conducted a descriptive numerical summary and categorized the included records to summarize the data. The descriptive summary included the total number of records, year of publication, and region of focus. Records categorized as “global” referred to studies that generated findings for the global evidence base without focusing on a specific country. We classified studies focused on individual countries according to the World Bank's country classification by income level.^23^

**FIGURE 1:**
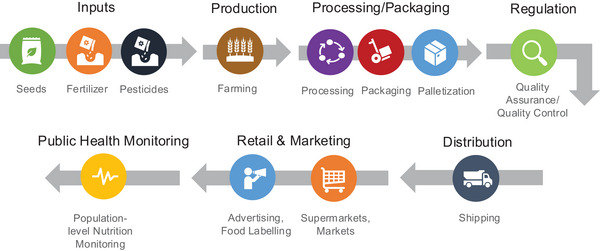
Food value chain. Biofortification starts with nutrient‐enriched seeds and inputs, while large‐scale food fortification begins during processing and packaging.^24^

To summarize data, we categorized records based on the phase of the food value chain they addressed (see Figure 1).^24^ We defined the food value chain as all the activities necessary to bring farm products to consumers (also known as farm to fork), including agricultural inputs, food production, food regulation, food retail and marketing, and public health monitoring.^25^, ^26^ We noted whether the study mentioned LSFF or biofortification.

We applied Ruthie Musker's framework for categorizing the types of data referenced in the studies (see Table 1) and validated this framework through internal consultation with the research team.^27^ We believe that this framework was the most relevant for categorizing the types of big data sources along the food value chain, both in records that mention food fortification and those that do not. Within big data analytics, we categorized records into four levels: descriptive analytics (what happened), diagnostic analytics (why it happened), predictive analytics (what is likely to happen), and prescriptive analytics (based on what is likely to happen, what are the best options for the way forward) (see Table 2).^28^

**TABLE 1:** Types of big data in agriculture and nutrition.^27^

Source data type	Definition
Remote sensing	Data collected from a distance (sensors, aerial/nonsatellite, satellite)
Computer vision, image analysis	Field of artificial intelligence (AI) that enables computers to derive information from large datasets of images or videos
Farm equipment and robotics	Farm equipment equipped with Global Positioning System (GPS), guidance systems, or crop‐specific sensors for planning, monitoring, analysis, and planning. Also known as “smart farming” and “precision agriculture”
Mobile phones through social media and crowdsourcing	Large‐scale collection and dissemination of data collected through mobile phones
Research	Any type of research data collected by industry, government, or academia, including market research collected by private sector companies on trade, marketing, wholesaling, retailing, and online sales or massive research datasets drawing from multiple diverse databases

**TABLE 2:** Types of big data analytics.

Big data analytics type	Definition	Use case example
Descriptive	Condenses data into smaller, more useful points of information to answer “What happened? Where? When?”	Using simple data dashboards to monitor soil health and water and fertilizer use or the coverage of large‐scale food fortification interventions
Diagnostic	Analyzes the patterns in historical data to understand “Why did it happen?”	Identifying the attributes most highly correlated with customer retention at an organic supermarket
Predictive	Transforms recent and historical data to determine “What is likely to happen?”	Forecasting crop yield or the impact of fortified food products on the prevalence of micronutrient intake
Prescriptive	Links predictive analytics with a prescription for “What should be done?”	Presenting options on which crops to plant, when to plant them, and the proper soil nutrients required to optimize yields or which large‐scale fortification policy option to implement in a specific context

## RESULTS

A total of 30,101 peer‐reviewed records met our eligibility criteria. After filtering duplicates, we identified a total of 18,439 potentially relevant records. Following the screening of titles and abstracts of all potentially eligible peer‐reviewed records, we excluded those that were irrelevant to the research question (*n* = 16,503). Additionally, we excluded peer‐reviewed records if no full text was available or if the full text was not in English (*n* = 92).

For a detailed screening, we obtained 1844 full‐text records in English. The reviewers carefully read each of the 1844 records, ultimately excluding 186 records from the study as they were not relevant to the research question. The final remaining number of peer‐reviewed records included in our study was 1658.

For the gray literature search, we retrieved 326 records. We screened the titles and abstracts of these records, removing duplicates (*n* = 32) and those that were not relevant to the research question (*n* = 265). We conducted a full‐text screening of 29 records and removed those that did not meet the eligibility criteria (*n* = 9). The final number of gray literature records from our targeted search was 20.

**FIGURE 2:**
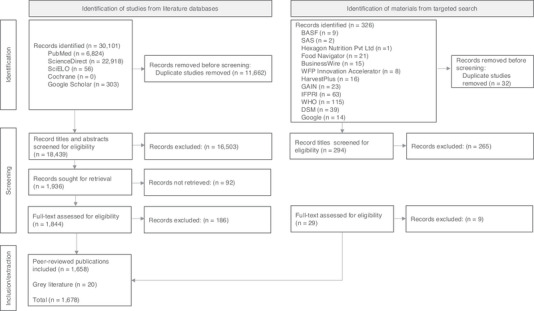
Papers included and excluded in the scoping review (PRISMA).

We included a total of 1678 records in the analysis. A PRISMA flow diagram is presented in Figure 2 to report the search screening process visually.

**FIGURE 3:**
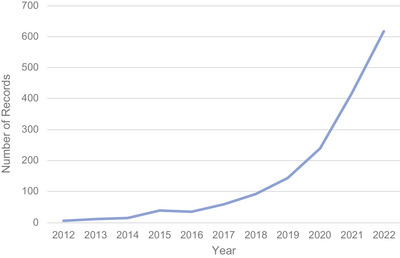
Increase over time in peer‐reviewed literature focused on agriculture‐ or nutrition‐related big data.

Our results reveal a large, diverse, and rapidly growing body of published records on the utilization of big data in the agriculture and nutrition field. The data showed that the total number of records published between 2017 and 2022 (*n* = 1571) is nearly 15 times higher than between 2012 and 2016 (*n* = 107), as illustrated in Figure 3.

Across the 10‐year period, 28 records (peer‐reviewed literature, *n* = 17; targeted search, *n* = 11) mentioned LSFF or biofortification. All were published between 2018 and 2022, with the following distribution: 2018 (*n* = 1), 2020 (*n* = 6), and 2022 (*n* = 15). “Mentioned” indicates that these records included the terms “food fortification” or “biofortification” at least once in their content. Of these, two records referenced both terms, 15 referring only to LSFF, and 11 referring only to biofortification.

We categorized the records by country and region of focus. Of the records with a focus on a specific country (*n* = 1027), 34.2% (*n* = 351) were located in East Asia and the Pacific, followed by 21.7% (*n* = 223) in South Asia and 14.5% (*n* = 149) in Europe and Central Asia. The distribution across the remaining regions was as follows: North America (11.1%, *n* = 114), Africa (10.2%, *n* = 105), Latin America and the Caribbean (5.6%, *n* = 58), and the Middle East and North Africa (2.7%, *n* = 27). By country, the highest number of records were focused on China (24.8%, *n* = 255), followed by the United States (10.9%, *n* = 112) and India (10.4%, *n* = 107).

**FIGURE 4:**
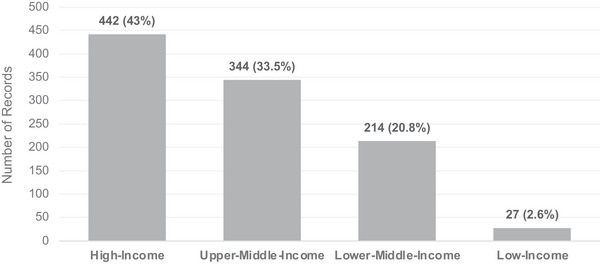
Records with a country focus by income group.

Among the records that mentioned LSFF or biofortification, two focused on Africa, two on Brazil, four on India, one on Indonesia, and the remainder had a global focus (*n* = 18) or a focus on Europe as a region (*n* = 1). When considering income groups, as presented in Figure 4, most records were focused on high‐income countries (47.9%, *n* = 819) and upper‐middle‐income countries (22.6%, *n* = 386). Within the low‐income group, Ethiopia accounted for most records, with 13 out of 27 (48.1%).

The records were published in 386 peer‐reviewed journals, nine online reports, four online news releases, and two blog posts on websites. Within the peer‐reviewed journals, the records covered several different scientific areas: agriculture (29.9%, *n* = 414), biology (13.4%, *n* = 223), engineering (13.1%, *n* = 218), information and communication technologies (12.3%, *n* = 205), chemistry (12.2%, *n* = 202), earth and environmental sciences (4.7%, *n* = 78), biomedical research (4.2%, *n* = 70), enabling and strategic technologies (2.9%, *n* = 49), clinical medicine (1.3%, *n* = 21), social sciences (0.8%, *n* = 14), economics and business (0.8%, *n* = 14), mathematics and statistics (0.5%, *n* = 8), physics and astronomy (0.5%, *n* = 8), public health and health services (0.4%, *n* = 6), psychology and cognitive sciences (0.3%, *n* = 5), built environment and design (0.2%, *n* = 4), and communication and textual studies (0.1%, *n* = 1). One hundred twenty‐two records (7.3%, *n* = 121) were published in journals without a scientific classification, meaning the journals do not have an overarching specific scientific classification (e.g., *PLoS One*).

When considering the types of big data sources across the records (Table 3), several examples showed that authors referenced two or more data types within a single analysis. For example, remote sensing data was often combined with other data collected for research to provide a more comprehensive analysis. Remote sensing data refers to data collected by in situ (subsurface) sensors (small‐scale, stationary, and attached to the earth, such as weather or water quality sensors), aerial/nonsatellite sensors (medium‐scale, mounted on aircraft such as Unmanned Aerial Vehicles or drones), and satellite sensors (large‐scale, mounted on satellites).^29^ Across all records, remote sensing data was the most commonly mentioned type (53.8%, *n* = 902), including records that mentioned LSFF or biofortification (42.9%, *n* = 12) and those that did not (53.9%, *n* = 890).

**TABLE 3:** Distribution of big data types across food value chain dimensions.

	Mentions LSFF or biofortification^a^		No mention of LSFF or biofortification^b^		Total mentions^c^	
Type	*n*	% within mentions	*n*	% within mentions	*n*	% within mentions
Remote sensing (sensors, aerial/nonsatellite, satellite)	12	42.9%	890	53.9%	902	53.8%
Computer vision, image analysis	3	10.7%	504	30.5%	507	30.2%
Farm equipment and robotics	9	32.1%	161	9.8%	170	10.1%
Mobile phones through social media and crowdsourcing	4	14.3%	61	3.7%	65	3.9%
Research	22	78.6%	645	39.1%	667	39.7%

^a^ Records with a mention of LSFF or biofortification (percentages calculated from a total of 28 articles).

^b^ Records without a mention of LSFF or biofortification (percentages calculated from a total of 1650 articles).

^c^ Total mentions across all articles (percentages calculated from the full dataset of 1678 articles).

When considering the type of big data analytics used, 44% (*n* = 739) employed descriptive analytics, typically for crop or environmental dashboards or retrospective descriptive studies, soil mapping, or food safety monitoring (Table 4). Predictive analyses were the next most common type of big data analytics used (40.3%, *n* = 676), generally for crop yield, crop stress (e.g., drought tolerance), food security, or shelf‐life projections.

**TABLE 4:** Distribution by type of big data analytics.

	Mentions LSFF or biofortification^a^		No mention of LSFF or biofortification^b^		Total mentions^c^	
Type	*n*	% with mentions	*n*	% within mentions	*n*	% within mentions
Descriptive	15	53.6%	724	43.9%	739	44.0%
Diagnostic	7	25.0%	388	23.5%	395	23.5%
Predictive	9	32.1%	667	40.4%	676	40.3%
Prescriptive	4	14.3%	148	9.0%	152	9.1%

^a^ Records with a mention of LSFF or biofortification (percentages calculated from a total of 28 articles).

^b^ Records without a mention of LSFF or biofortification (percentages calculated from a total of 1650 articles).

^c^ Total mentions across all articles (percentages calculated from the full dataset of 1678 articles).

**FIGURE 5:**
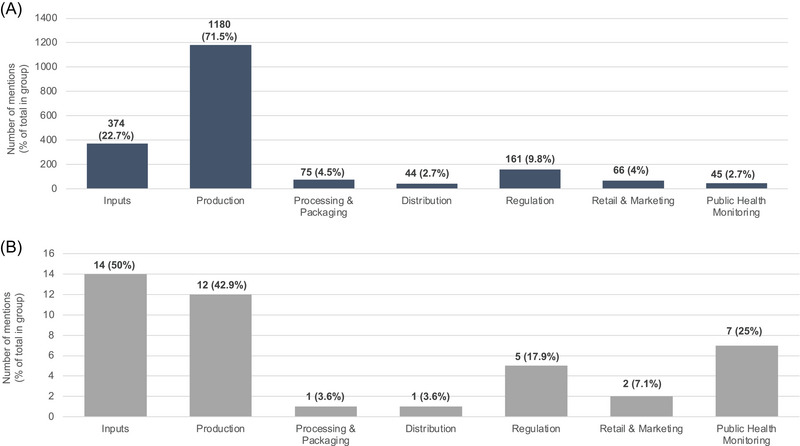
Distribution of mentions across food value chain dimensions: (A) records without a mention of LSFF or biofortification (proportions calculated from a total of 1650 records), and (B) records with a mention of LSFF or biofortification (proportions calculated from a total of 28 records). Percentages for each dimension represent the proportion of mentions within each group, not the overall dataset.

Across the food value chain dimension (Figure 5), production represented the largest share of mentions (60%, *n* = 1192), followed by inputs (19.5%, *n* = 388). The distribution of mentions varied between records with and without references to LSFF or biofortification. Records mentioning LSFF or biofortification focused more on public health monitoring (16.7%, *n* = 7) than those without such mentions (2.3%, *n* = 45).

From the 1678 records retrieved, 28 explicitly mentioned LSFF or biofortification, and we identified eight distinct use cases as directly relevant to these interventions (Table 5). These applications include using blockchain and IoT to build supply chain traceability systems (regulation), leveraging robotic technologies to optimize bread production processes (production and processing), and employing machine learning to predict consumer preferences for fortified products (retail and marketing). Other records highlight transferable innovations in agriculture and nutrition that could be adapted to food fortification, such as nutrition early warning systems and large‐scale dietary data analysis for public health monitoring.

**TABLE 5:** Big data use cases from records mentioning LSFF or biofortification.

Use case	Food value chain dimension	Type of big data	Type of big data analytics	Is the use case currently relevant for informing food fortification or biofortification programs?	Citation
Nutrition Early Warning Systems (NEWS) or hunger mapping systems that use machine learning to aggregate and analyze satellite imagery and traditional data to provide ongoing surveillance of nutrition threats and options for nutrition interventions	Public health monitoring	Remote sensing	Descriptive	No	93
Gather, organize, and analyze near real‐time information on the impact of the COVID‐19 pandemic on food and agriculture value chains, food prices, food security, and interventions	Distribution, public health monitoring	Research	Descriptive	No	93
Find patterns of large nutrition datasets otherwise recognizable to humans that can predict or model outcomes	Public health monitoring	Research	Predictive	No	93
Quickly diagnose patients with nutritional conditions using machine learning in an app (e.g., malnutrition based on anthropometric data inputs)	Public health monitoring	Research	Descriptive	No	93
Model human sensory perception and predict consumer preferences for food and beverage products to make healthy foods more palatable and more widely accepted	Retail and marketing	Research	Predictive	Yes	94
Apply blockchain and IoT to build a supply chain traceability system for fortified products	Regulation	Research	Descriptive	Yes	95, 96
Integrate robotic technologic intensifiers to automate and optimize bread production for fortification	Production and processing	Remote sensing	Prescriptive	Yes	97
Use artificial intelligence to analyze anemia prevalence	Public health monitoring	Research	Descriptive	Yes	98

## DISCUSSION

### What big data is available for LSFF or biofortification?

For centuries, data has been the basis for empirical decisions in every segment of the food value chain, from agriculture to production, logistics, and distribution. However, these data were often only collected locally at smaller units, rarely digitized, and seldom made available by stakeholders to be integrated into big data. Today, all segments of the food value chain generate and manage massive amounts of structured and unstructured data. The application of big data in the food industry offers significant opportunities for optimization and innovation across the entire value chain, from farm to fork. By analyzing this wealth of information, stakeholders at every level—from farmers to consumers—can make more informed and efficient decisions.

Despite this potential, our findings suggest that LSFF and biofortification have yet to fully benefit from the big data revolution. While numerous innovations in agriculture and nutrition, such as blockchain for supply chain traceability and machine learning for consumer insights, have emerged, their direct application to LSFF and biofortification remains limited. Transferable technologies, such as nutrition early warning systems and dietary data analysis, could enhance decision‐making in LSFF and biofortification strategies; however, there is a clear need for more targeted exploration and integration of big data in this field. These gaps present an opportunity to bridge the divide and fully leverage big data's potential to advance fortification efforts.

Currently, only a handful of countries, including Cameroon and Palestine, utilize recent dietary intake and MND data to inform the design of LSFF programs.^30^ Further, data on the quality and coverage of ongoing LSFF programs are not routinely collected across countries, which limits decision‐makers’ ability to track progress and identify and address implementation challenges.^31^

Online management information systems, such as FortifyMIS and the Global Fortification Data Exchange (GFDx), provide valuable access to national program data for the nutrition research, programming, and policy community. However, these systems do not meet the criteria of big data. While FortifyMIS is a useful online tool for collecting and aggregating fortification monitoring data, it does not meet the criteria for big data as it primarily focuses on a limited number of data sources and is designed to benefit policymakers and industry stakeholders.^32^ Similarly, GFDx is an online analysis and visualization tool that aggregates data from 196 countries on five commonly fortified foods (maize flour, oil, rice, salt, and wheat flour) to demonstrate progress or alignment with related global targets and recommendations.^33^ To be considered a big data source, these systems would need to have a significantly larger amount of data from a wider variety of sources (volume), a high rate of data intake and processing to assess fortification efforts in real‐time (velocity), and incorporate a diverse range of data types (veracity).

### The use of big data along the food value chain

Stakeholders are increasingly utilizing big data along the food value chain to improve efficiency, productivity, and transparency.^34^, ^35^, ^36^ This scoping review revealed that over 80% of the records focused on input and production segments, particularly in agriculture and precision farming (Figure 5). These stages generate extensive datasets that inform decisions on crop health, soil conditions, and environmental factors. However, as shown in the scoping review, segments such as regulation and public health monitoring accounted for only a relatively small proportion of records—2.3% and 8.3%, respectively. This distribution reveals a significant gap in the application of big data within these critical areas.

Applying big data in processing, regulation, and public health monitoring could address key challenges in LSFF and biofortification, such as ensuring compliance, optimizing nutrient delivery, and monitoring public health outcomes. LSFF and biofortification are relevant to every dimension of the food value chain, from production to public health surveillance. However, only 28 of the 1678 records analyzed in this scoping review mentioned LSFF or biofortification. Despite the limited literature, there are clear opportunities to integrate big data more effectively across the value chain to enhance LSFF and biofortification efforts. Below, we review the use of big data along the food value chain as described in the records included in this scoping review and highlight opportunities for the LSFF and biofortification sector.

#### Inputs

In agriculture, stakeholders leverage big data to improve efficiency and productivity while also minimizing waste and reducing environmental impact. This scoping review highlights how smart farming and precision agriculture approaches generate large amounts of agricultural data that can be classified as big data.^37^, ^38^, ^39^, ^40^ Sophisticated farm equipment and robotics, equipped with GPS guidance systems and sensors for weed management, utilize remote sensing data from satellites and ground‐based systems.^41^ Robotic arms on larger farms use big data algorithms to harvest fruits and assess optimal ripeness and nutritional value.^42^, ^43^ Autonomous robots have also been trained through machine learning to generate data on soil samples, such as mapping the potential hydrogen (pH) and mineral content of soils in real‐time.^44^ In crop monitoring and risk management, farmers use satellite data to monitor crop growth, health, and environmental factors, such as weather patterns or pests, that can impact crop yield and quality.^45^, ^46^, ^47^

Many existing enabling technologies also support predictive analytics and supply chain optimization in the agricultural sector. Predictive analytics and supply chain optimization use big data to analyze weather patterns, market trends, and other factors to predict future crop yields and prices.^48^, ^49^, ^50^, ^51^ This information can help farmers make informed decisions about planting, harvesting, and marketing crops. For example, insights from farm‐level big data models may indicate that, due to specific weather patterns, raw materials may have higher levels of toxins (e.g., aflatoxin), which can lead farmers to create appropriate mitigation measures.^52^

Using big data in agriculture presents an opportunity to strengthen inputs for LSFF or biofortification. For example, understanding the natural micronutrient content of staple crops can help determine the appropriate amount of premix to add during food production, ensuring optimal fortification levels. Furthermore, decision‐makers can apply predictive analytics and supply chain optimization to assess weather patterns, market trends, and other variables to forecast crop yields and nutrient content—enabling more strategic decisions about crop selection and biofortification efforts. The increased use of mobile phone technology also allows smallholder farmers to access big data and receive recommendations on good agricultural practices for biofortified crops.

#### Processing and packaging

To achieve optimized production processes, increased efficiency, and reduced waste, food processing and packaging processes are optimized by identifying inefficiencies and opportunities for improvement.^53^, ^54^, ^55^ Machine performance, production time, or inventory levels can provide relevant information. Traceability and quality control throughout the production process, from raw materials to finished products within a given production unit or from farm to fork, provide regulators with information to identify and prevent quality issues before they become a significant problem, or improve production processes.^53^, ^56^, ^57^ Improving food safety helps identify potential safety issues during processing and packing and prevents them from occurring.^58^ Investigators can use machine learning and big data from social media or public health reports to rapidly identify the source of contamination in the event of a foodborne outbreak.^59^

Big data could be a powerful tool for optimizing the processing and packaging of fortified and biofortified food products. By analyzing machine performance, production time, and inventory levels, big data can identify opportunities for improvement, resulting in increased efficiency, reduced waste, and potentially lower costs. Additionally, big data can enhance the premix process by evaluating the quality and nutrient content of raw materials, refining formulations and dosages, and ensuring traceability from raw materials to finished products.

#### Regulation

The regulatory segment of the food value chain is concerned with the quality and safety of food. Regular quality assurance audits are a significant source of data for this dimension of the food value chain. The audits are bolstered by the continuous collection of food quality and safety data generated by the stakeholders under investigation (using certified methods and equipment), which regular tests performed by reference laboratories may verify. Developing new solutions for rapid and cost‐effective on‐site testing methods can generate highly comprehensive data sets on food quality and safety.^35^, ^60^, ^61^ These data, combined with real‐time monitoring of environmental conditions such as temperature and humidity during storage and transportation, can form the basis for a meaningful analysis to improve compliance and the monitoring process. A prerequisite for successful monitoring is the ability to trace products from farm to consumer.^62^ Two other aspects are consumer feedback, which helps identify potential quality issues and improve overall product quality and regulatory compliance.^63^, ^64^, ^65^ The information gained from tracking food safety and quality data helps companies comply with food safety regulations, avoiding costly penalties and legal issues.^66^

In the case of fortified or biofortified food products, decision‐makers can use big data to monitor and improve compliance with regulatory standards. For example, they can track the quality and safety of nutrient content and verify that labeling accurately reflects what the product contains. In relation to regulation, big data can also help predict potential safety issues during the processing and packaging of fortified and biofortified products, thereby preventing them from occurring. By analyzing data generated from on‐site testing methods and real‐time monitoring of environmental conditions, stakeholders can develop a comprehensive understanding of the safety and quality of these products throughout the entire value chain, from farm to consumer. This information can help regulators ensure that fortified and biofortified food products meet safety and quality standards, ultimately benefiting public health.

#### Distribution

Big data in food distribution is most relevant to transportation logistics.^67^ Companies use big data approaches to optimize their supply chains, reduce costs, and increase customer satisfaction. For route optimization and real‐time tracking, distributors utilize big data to analyze traffic patterns and weather conditions, thereby optimizing delivery routes and preserving temperature‐sensitive food products.^68^ Maintaining consistent temperatures during shipping is particularly important for fortified foods containing vitamins that are sensitive to degradation. Real‐time tracking data allows companies to monitor the progress of their shipments.^69^ Additional value comes from inventory management, demand forecasting, and customer analytics data. These three aspects help ensure that the right products are available at the right time, improving customer satisfaction and reducing waste due to overstocking.^70^

Big data also enables more transparent tracking of fortified and biofortified food products throughout the supply chain, helping stakeholders identify delays, shortages, and inefficiencies in distribution. For example, companies can use big data analytics to track the movement of fortified food products across the supply chain, from production facilities to warehouses to distribution centers to retailers. With real‐time tracking, companies can monitor the temperature and humidity conditions of their shipments and intervene quickly to prevent spoilage or degradation of nutrients. By optimizing inventory levels and improving demand forecasting, companies can also ensure that fortified and biofortified products are available when and where needed, reducing waste and improving consumer access.

#### Retail and marketing

Utilizing big data in the retail grocery industry aims to enhance supply chain efficiency, balance consumer demand and supply, improve the customer experience, and optimize store operations.^36^, ^71^, ^72^ This is especially important for perishable goods and heat‐sensitive ingredients, such as vitamins, where maintaining proper temperatures during storage is critical. Managers can monitor these conditions using smart sensors and machine learning algorithms.^73^, ^74^ Retailers can improve supply chain efficiency, avoid stockouts, reduce waste, and ultimately lower costs through effective inventory management and supply chain optimization, which includes leveraging sales data, seasonal trends, supplier performance, and transportation routes.^75^, ^76^, ^77^, ^78^ Regulators can use big data on the geographical distribution of fortified foods to assess whether fortification levels need to be adjusted to meet population needs. Big data can also inform price optimization, personalization, and customer experience, helping retailers stay competitive while maximizing profits and enhancing the shopping experience through personalized recommendations based on consumer purchase history and preferences.^79^, ^80^

Big data can support the effective delivery of fortified and biofortified foods by helping identify where needs are greatest and ensuring consistent product availability. Retailers and supply chain actors can use demographic and geographic data to pinpoint communities with high rates of MNDs and prioritize those areas in their distribution plans. Real‐time data on inventory and sales can also help maintain steady supplies in these locations, improving access to essential nutrients.

#### Public health monitoring

Big data analytics can significantly enhance public health nutrition monitoring by providing valuable insights into the nutritional status of populations, identifying nutritional deficiencies and risk factors, and evaluating the effectiveness of interventions. This approach involves leveraging diverse datasets, including health records, dietary intake surveys, and food composition databases, to develop evidence‐based policies that address the nutrition needs of populations.

The availability of reliable, population‐level LSFF data can help catalyze public health action, refine program interventions, and contribute to greater cost‐effectiveness and safety of public health programs. In Guatemala, for example, national‐level data on the prevalence of vitamin A deficiency led to research on vitamin A fortification, resulting in the introduction of an evidence‐based national sugar fortification program alongside other supportive interventions.^81^ Although the use of sugar as a fortification vehicle is debated due to concerns about rising noncommunicable diseases, sugar fortification programs have been effective in improving vitamin A status in some populations and have also contributed valuable data to inform program design and monitoring. Machine learning algorithms and big population datasets have been used in several countries to identify risk factors for malnutrition, predict nutritional outcomes, and design nutrition interventions, including LSFF programming.^82^, ^83^

### How can big data analytics contribute to decision‐making and sustainability in LSFF or biofortification?

Two key data sources from stakeholders along the food value chain can inform LSFF and biofortification decision‐making. The first is large‐scale data collected for other purposes, such as precision agriculture, production, regulation, and public health monitoring. These datasets (e.g., weather, crop quality, and consumer preferences) often meet big data criteria due to their high volume, velocity, variety, veracity, and value. The second is process‐specific data generated during LSFF or biofortification activities, including quality assurance/quality control or local environmental data. While some of this data may qualify as big data, much does not, highlighting the need to assess where big data can provide the greatest return on investment (ROI) in LSFF or biofortification decision‐making. By identifying high‐impact areas, stakeholders can maximize economic returns while enhancing the sustainability of LSFF and biofortification programs through more efficient resource use, reduced waste, and improved public health outcomes.

Big data, in and of itself, does not hold any inherent value; instead, it is the insights and actions derived from these insights that yield returns. Like other industries, the implementation of big data in the food sector has the potential to generate significant economic savings. However, this potential benefit comes with cost challenges, including the hardware required for data collection and analysis. It is important to note that these challenges are less critical in the larger (and economically stronger) food production market than the relatively tiny LSFF and biofortification segment. Therefore, to determine the role of big data in LSFF and biofortification, it is crucial first to define the appropriate points in the food value chain that would benefit from big data analytics.

In 2021, the global food market generated approximately $8.27 trillion in revenue.^84^ In 2020, experts valued the global food fortification market at $69.8 billion.^85^ North America dominated the market with over 33% of the total share, followed by Europe and Asia Pacific. These regions have a higher demand for fortified foods and supplements due to greater awareness about the benefits of fortification and higher purchasing power compared to developing countries. In LMICs, government‐led initiatives primarily drive the market, with LSFF or biofortification initiatives aimed at addressing regional or national malnutrition and MNDs. These data clearly illustrate the significant economic and regional disparities in the food fortification market between high‐income versus LMICs. In such a small market, investing in big data‐driven analytics for LSFF or biofortification must be carefully evaluated to ensure cost‐effectiveness and sustainability.

### What are the challenges of using big data along the food value chain?

The use of big data in the food value chain poses significant challenges, particularly in the niche market of LSFF and biofortification. One of the primary challenges is the lack of skilled human resources capable of collecting, analyzing, and disseminating big data to a diverse audience. This is particularly true in LMICs where funding for data science training is limited, and brain drain exacerbates talent shortages.

Cultural barriers, such as resistance to change, a lack of trust, and siloed data, also hinder the adoption of big data. Resistance to new technologies necessitates effective change management, while siloed data—caused by rigid organizational structures and poor collaboration—limits comprehensive analysis. Trust issues among stakeholders, including government entities and industry players, further restrict data sharing due to concerns over intellectual property, competition, and confidentiality. This results in inefficiencies, redundant testing, and increased operational costs. Addressing these cultural barriers requires collaboration, transparency, and trust‐building.

Ethical challenges include privacy concerns, data ownership, and the right to use big data.^86^, ^87^, ^88^ Individuals across the food value chain—farmers, manufacturers, and consumers—may be unaware that their data is being collected or have not consented to its use. Transparency and accountability are essential to addressing these concerns and fostering trust.

Technical challenges include data quality, security, governance, integration, and analysis of big data sources. It is crucial to have the appropriate digital infrastructure with significant storage and processing capabilities to handle the volume and velocity of data. Integrating individual subsets of data into big data can be difficult as data often originates from disparate systems, is in various formats, and is unstructured. Furthermore, these data may have quality problems, such as data errors, missing information, and inconsistencies that require significant time and effort to clean, extract, integrate, and analyze the final big dataset.^89^

To address these challenges, organizations need to have robust data governance practices in place, along with standardized processes, to ensure compliance with and adherence to relevant privacy regulations.^90^ They must ensure that the rights of individuals are protected and that the benefits of big data are shared equitably.^91^ Investments in data governance, data literacy, and programs that build awareness of the value and impact of big data to stakeholders along the food value chain are needed. These efforts can help overcome technical, cultural, and ethical barriers, enabling the broader adoption of big data approaches within the food system, particularly in LSFF and biofortification.

Despite promising applications, our review identified several critical knowledge gaps that limit the advancement and use of big data in LSFF and biofortification. First, feasibility remains a major challenge, especially in contexts where digital infrastructure, data systems, and institutional capacity for implementing big data approaches are still developing. Second, the economic viability and cost‐effectiveness of big data approaches remain poorly documented, making it difficult for governments and donors to justify or prioritize such investments. Third, few studies assess the technological readiness of the proposed tools, including their scalability, interoperability with existing systems, and ability to provide real‐time insights. Fourth, there is limited evidence on how decision‐makers are translating big data into programmatic action. Finally, equity considerations are often overlooked, with little exploration of whether big data enhances access to fortified foods for vulnerable populations or risks exacerbating existing disparities. Future research should aim to fill these gaps to guide more effective and inclusive implementation of big data strategies.

### Limitations

This study has six main limitations. First, a selection bias may be present, as the search only retrieved records written in English, thereby excluding records written in other languages. Second, the search only retrieved freely available (open access) records. Including gated records could have uncovered additional relevant studies. Third, our search methodology's explorative approach and broad focus make retrieving and screening all relevant literature challenging. Fourth, the breadth of the research question may have inevitably affected the depth of our analysis and may prevent generalization. Fourth, the lack of standard terminology in big data may have limited the effectiveness of our keyword selection, leading to the omission of some relevant literature. Finally, since LSFF or biofortification is often integrated as part of government programs in multiple settings, it is possible that our review may be missing unpublished public sector examples and, therefore, may not include perspectives from government stakeholders and management systems.

Selection biases are a risk in any literature review, as the number of databases a research team can feasibly search is inherently limited. To minimize this bias, we focused on major databases that encompass a wide range of interdisciplinary research in agriculture, nutrition, food science, computer science, and engineering. Additionally, we supplemented our search by exploring gray literature through a targeted approach, which likely enhanced the comprehensiveness of our review.

The third and fourth limitations are inherent to the scoping review method, which is “more narrative in nature,”^92^ aims to provide a “richly informed starting point for further investigations,”^92^ and is typically not presented through descriptive statistical analysis. Still, we believe the scoping methodology was the best approach due to the broad focus of our research question. To address the final limitation, we incorporated input from nutrition experts to refine our search terms and keywords. Future improvements could involve conducting more expert interviews and surveys to gather additional insights and identify relevant literature we may have missed with our selected keywords.

## CONCLUSION

LSFF and biofortification are critical strategies to combat the global challenge of malnutrition. Economically, these interventions represent a relatively small market in LMICs, driven primarily by government and donor investments, which may limit the prioritization of data‐driven decision‐making. This scoping review revealed that most data relevant to LSFF and biofortification do not yet meet the criteria for big data. However, leveraging insights from big data applications in agriculture and food production could enhance LSFF and biofortification, particularly when the ROI justifies the cost.

Increasing the volume, velocity, variety, veracity, and value of data along the food value chain could strengthen data‐informed decision‐making, improve the evaluation of long‐term intervention impacts, and facilitate more systematic identification of programmatic bottlenecks. For example, big data approaches can help integrate diverse datasets across production, distribution, and consumption systems. This integration enables more comprehensive analyses of where inefficiencies occur and how they affect the reach and effectiveness of fortified foods. While the potential benefits are considerable, challenges such as fragmented and partially interoperable information systems, as well as limited technical and institutional capacity, particularly in LMICs, persist in the LSFF and biofortification sectors, hindering the processing and analysis of large‐scale datasets. Addressing these constraints will require sustained structural and economic investments across the public and private sectors.

This review also highlights several promising applications that may warrant further exploration through in‐depth analyses or systematic reviews. In particular, the use of big data for public health surveillance and regulatory compliance could strengthen fortification programs by enabling more timely monitoring of nutrient status and adherence to standards. Similarly, predictive analytics and supply chain optimization, already in use in adjacent sectors, could be adapted to improve LSFF and biofortification planning and responsiveness. These applications, along with a clearer understanding of feasibility, cost‐effectiveness, and equity implications, represent important priorities for future research and investment. These insights may support policymakers, donors, and implementers in identifying priority areas for investment, adaptation, and further study.

## AUTHOR CONTRIBUTIONS

F.W. and F.S. designed the study and developed the methods. F.W. and F.S. analyzed the data. F.W. wrote the first draft of the manuscript. All authors contributed to the data interpretation and revisions of the manuscript and read and approved the final manuscript.

## CONFLICT OF INTEREST STATEMENT

The authors have no conflicts of interest to declare.

## Supporting information



SUPPLEMENTARY FILE: APPENDIX S1

SUPPLEMENTARY FILE: APPENDIX S2

## Data Availability

The data that support the findings of this study are available from the corresponding author upon reasonable request.

## Footnotes

a Other papers part of a special virtual issue of *Ann NY Acad Sci* on Potential of Digital Technologies to Strengthen the Food Fortification Value Chain address topics related to the synthesis of a technical meeting on digital technologies, the use of digital technologies in fortification, digital technologies and the food value chain, and behavior change interventions, digital technologies, and fortified foods.
