# Supplementary material for: How big data analytics can strengthen large‐scale food fortification and biofortification decision‐making: A scoping review
Source: Ann N Y Acad Sci. 2025 Sep 10;1552(1):78–93. doi: 10.1111/nyas.70028 (PMC12576868; doi:10.1111/nyas.70028)
Supplement: Supplementary file 2 — SUPPLEMENTARY FILE: APPENDIX S2 [file NYAS-1552-78-s001.docx]

**SUPPLEMENTARY FILE: APPENDIX 2**

**Additional Information on the Search Strategy**

This section provides detailed information on the search strategy used to identify relevant materials for this scoping review. For comprehensive details on the search strategy and eligibility criteria, please refer to the “Materials and Methods” section of the manuscript.

We used a two-step approach to conduct the search strategy, described below:

***Step 1. Review of peer-reviewed literature***

The search strategy was designed to locate peer-reviewed research published in the English language only. There were no restrictions on the type of study design. Searches were conducted across six databases (PubMed, ScienceDirect, Cochrane Database of Systematic Reviews, SciELO, and Google Scholar) using the keywords described in Table 1. The searches in these databases searched the articles’ titles, abstracts, and keywords.

**Table 1.** Search terms for peer-reviewed literature

| **Search terms** |
| --- |
| ("Big Data" OR "Data Mining" OR "Datamining" OR "Data Driven" OR "Data Analytic*" OR "Machine Learning" OR "Artificial Intelligence" OR "Predictive Model*") AND ("Nutrition" OR "Diet" OR “Fortif*” OR "Biofortif*" OR "Micronutrient*" OR "Premix" OR Agriculture OR Farming OR “Food Distribution” OR “Food Marketing” OR “Food Monitoring” OR “Food Processing” OR “Food Quality” OR “Food Safety” OR "Food Regulation" OR “Food Storage” OR “Food Supply” OR “Food System*” OR “Food Traceability” OR “Food Value Chain*”) |

The number of extracted records is presented in Table 2. We retrieved the records from the first five pages of Google Scholar.

**Table 2.** Number of records retrieved by each search database

| **Database** | **Number of records retrieved** |
| --- | --- |
| PubMed | 6,824 |
| ScienceDirect | 22,918 |
| SciELO | 56 |
| Cochrane Database | 0 |
| Google Scholar | 303 |

***Step 2. Grey literature review***

In the second step, we manually added unique materials published on the websites of organizations working on food fortification or from a search of the standard Google search engine to capture records that may have been missed because they do not match our search terms. The following were searched:

- Relevant citations in peer-reviewed literature
- Targeted website searches, including:
  - Cargill
  - SAS
  - General Mills
  - Wilmar
  - AkzoNobel
  - Hexagon
  - BASF
  - Food Navigator
  - Businesswire
  - World Food Programme Innovation Accelerator
  - HarvestPlus
  - GAIN
  - IFPRI
  - WHO
  - GAIN Premix Facility-Approved List of Suppliers:
    - AQC Chem Lab (P) Ltd
    - Beijing Jinkangpu Food Science and Technology Co., Ltd.
    - Coalescence, LLC
    - DSM Nutritional Products AG
    - Evarom Gida
    - Global Calcium Private Ltd
    - Hexagon Nutrition Pvt Ltd
    - Manisha Pharmo Plast Pvt Ltd
    - Miavit Food GmbH
    - Mirpain Gida San. ve Tic. A.S.
    - Mühlenchemie / SternVitamin
    - NuTaste Food & Drink Labs Pvt Ltd
    - Nutralia S.R.L
    - Nutrition Supplies and Services (Ireland) Limited
    - PD Navkar Bio Chem Pvt Ltd
    - Piramal Pharma Ltd
    - Polen Un Ve Gida
    - Prinova Europe Ltd
    - Pristine Organics
    - Repco
    - Remidex Pharma Pvt. Ltd
    - Sudeep Nutrition Pvt. Ltd
    - The Wright Group
    - Ufuk Kimya Ilac San.Tic.Ltd.Stl
    - Vitablend Nederland BV
    - Wella Nutrologicals
- Standard Google search engine with keywords used for Google Scholar search in Step 1.
